# Reversible oxidative dimerization of 4-thiouridines in tRNA isolates†

**DOI:** 10.1039/d3cb00221g

**Published:** 2024-02-01

**Authors:** Larissa Bessler, Jonathan Groß, Christopher J. Kampf, Till Opatz, Mark Helm

**Affiliations:** a Institute of Pharmaceutical and Biomedical Sciences, Johannes Gutenberg University Mainz Staudingerweg 5 55128 Mainz Germany mhelm@uni-mainz.de; b Department of Chemistry, Johannes Gutenberg University Mainz Duesbergweg 10–14 55128 Mainz Germany

## Abstract

The occurrence of non-canonical nucleoside structures in RNA of biological or synthetic origin has encountered several recent boosts in attention, namely in the context of RNA modifications, and with an eye to RNA vaccines. New nucleoside structures introduce added functionality and function into biopolymers that are otherwise rather homogenous in their chemical structure. Here, we report the discovery of a presumed RNA modification that was identified by combination of liquid chromatography–tandem mass spectrometry (LC–MS/MS) with stable isotope labelling as a dimer of the known RNA modification 4-thiouridine (s^4^U). The disulfide-linked structure, which had previously been synthetically introduced into RNA, was here formed spontaneously in isolates of *E. coli* tRNA. Judicious application of stable isotope labelling suggested that this presumed new RNA modification was rather generated *ex vivo* by oxidation with ambient oxygen. These findings do not only underscore the need for caution in the discovery of new RNA modifications with respect to artifacts, but also raise awareness of an RNA vulnerability, especially to oxidative damage, during its transport or storage.

## Abstract 2

In search for new RNA modifications in *E. coli* tRNA we elucidated a disulfide-bridged dimer of 4-thiouridine which was identified as an *ex vivo* artifact and is formed during sample handling in the presence of ambient oxygen.

## Graphical abstract

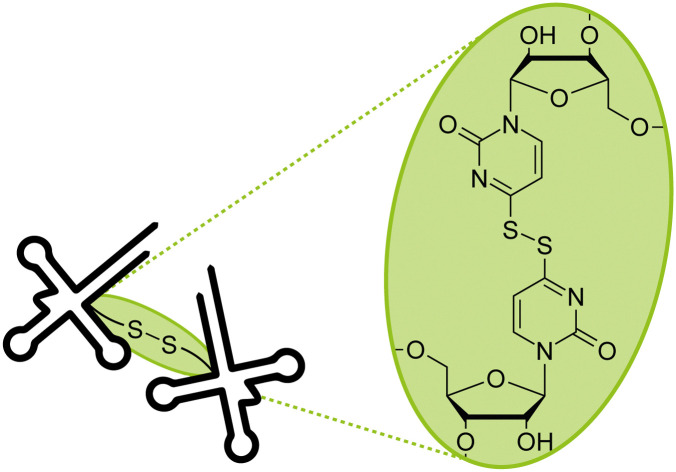

## Introduction

Non-canonical nucleoside structures in native RNA comprise some 170 modified ribonucleoside structures as part of so-called epitranscriptomes, and their chemical diversity is thought to enable control and fine-tuning of a plethora of cellular processes.^1–3^ Recent developments have shown the tremendous importance even of otherwise rare modifications such as 1-methylpseudouridine (m^1^Ψ) in biomedical applications.^4–7^ New biological structures are continuously being discovered, especially in transfer RNA (tRNA) which harbours not only the greatest number of modifications per molecule but also the greatest structural variety of modifications among different RNA species.^8–12^ Beyond RNA modifications that are enzymatically installed on purpose, the structure of ribonucleosides can also be altered *in vivo* in the context of RNA damage upon exposure to endogenous and exogenous agents, resulting in alkylated or oxidized nucleosides which hinder RNA in its proper functioning and are associated with a variety of pathophysiological conditions.^13,14^ Yet further, in addition to structural alterations resulting in both, RNA modifications and damaged nucleosides that are introduced *in vivo*, the storage and handling of RNA can also lead to structural aberrations *ex vivo*. For example, new non-canonical nucleosides were found to be formed *in vitro* during RNA isolation, storage or sample preparation.^15–17^ Improved instrumentation and lowered detection limits have enabled the detection of ever more nucleoside species. Elucidation of such structures is important to sort genuine artifacts from biological or chemical damage. A very recent example illustrates the importance of the respective analytical chemistry of new structures in quality control of medicinal RNA.^18^ Many artifacts resulting from deamination, Dimroth rearrangement, oxidation or hydrolysis may also occur as genuine modifications in native RNA whereas a minor fraction consists of non-natural structures, which were shown to be generated *in vitro*.^16,19,20^ The occurrence of such structures may not only lead to wrong appreciation of the abundance of known modifications but can also confuse and impede the LC–MS-based identification of new RNA modifications.^16,21^ More important to the wider community, certain structures can also impair functionality of the sample in downstream experiments or applications.^22^ One example scrutinised in this context is 8-oxo-guanine (8-oxo-G), the flagship among oxidative lesions in both DNA and RNA, which originates from guanine both *in vivo e.g.* resulting from reaction with reactive oxygen species (ROS) and *in vitro*, either by addition of oxidizing agents or as a consequence of sample preparation and storage.^17,23–25^ As a case in point, 8-oxo-G sites may serve as an example of the effects of RNA lesions on their functionality. Thus, in mRNAs used in *in vitro* translation systems or being administered to living cells, 8-oxo-G residues were demonstrated to not only reduce translational efficiency and induce ribosome stalling,^22,26,27^ but also lead to rapid RNA degradation by surveillance mechanisms.^28–32^ Especially in view of the high therapeutic potential of mRNA as showcased in the form of vaccines during the SARS-CoV-2 pandemic, it is important to consider the effects of manufacturing, long-term storage, and contact with atmospheric oxygen on the integrity of an RNA sample. A pertinent example is the recent discovery of lipid adducts of vaccine mRNA upon extended storage accompanied by significant reduction of translation efficacy.^18^ The above examples underline the necessity to improve detection and structure elucidation techniques for new nucleoside species, and to aim for a comprehensive understanding of all processes contributing to the chemical space of nucleoside species encountered *in vivo* and *in vitro*, be their origin of biological or synthetic nature.

We have scrutinised the nucleoside content of tRNA isolates from *E. coli* for novel structures using a specifically designed LC–MS regimen termed neutral loss scan (NLS, *vide infra*), and previously reported on two newly identified modification types, along with three dozen candidates of yet unidentified structures.^11,33,34^ Here we report on the pursuit of a group of four unusual LC–MS signals, which we found to originate from what was initially a putatively new RNA modification. Its structure turned out to be an oxidatively damaged dimeric form of s^4^U, linked *via* a disulfide. Isotope labelling experiments argue for formation of this nucleoside structure *in vitro*, namely upon storage in the presence of ambient oxygen. This constitutes first experimental evidence hinting at possible storage artifacts in RNA samples containing sulphur, particularly s^4^U, *e.g.* in correspondingly labelled samples intended for bioconjugation, RNA enrichment, photo-crosslinking or RNA lifetime analysis.^35–40^

## Results and discussion

**Fig. 1:**
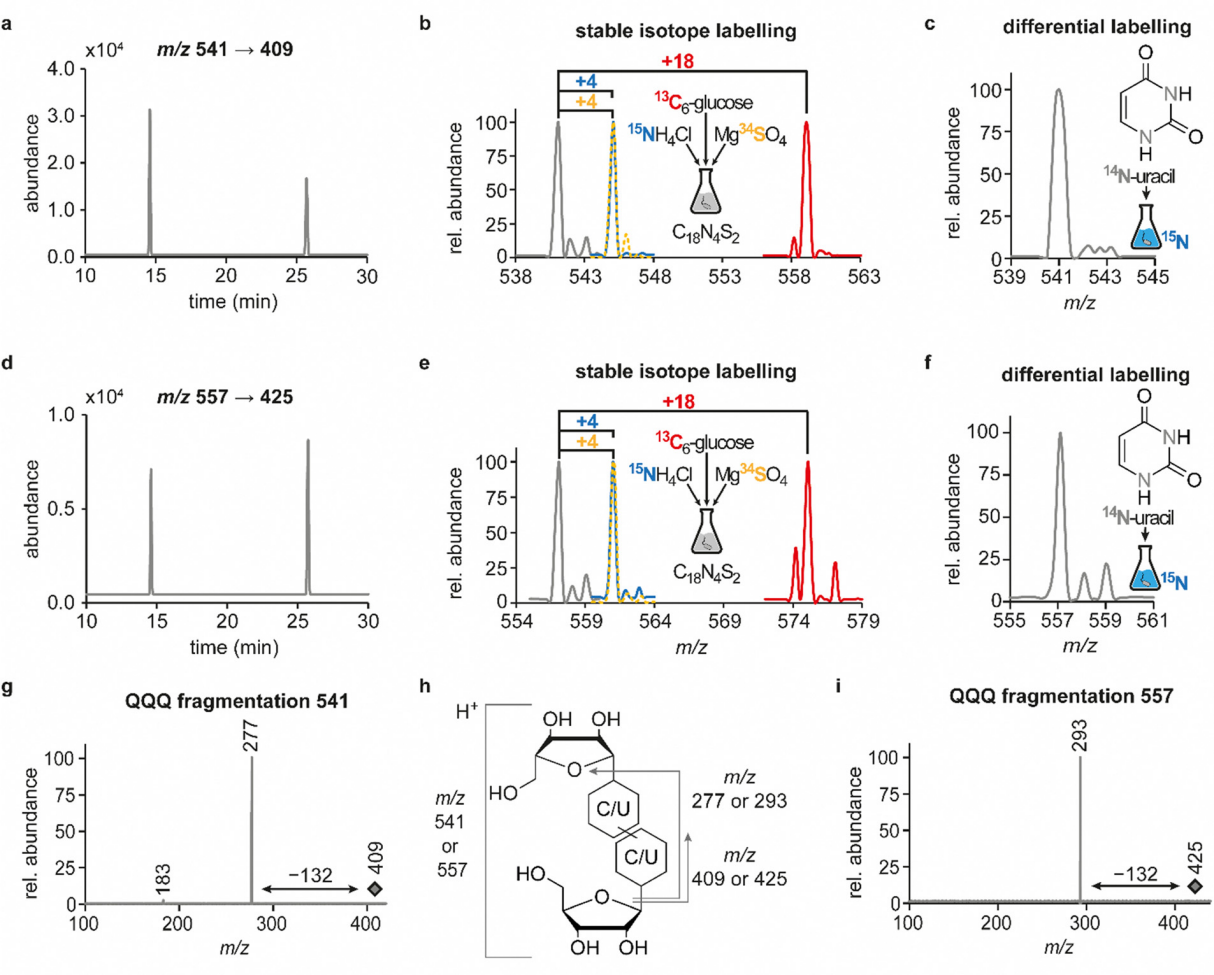
Structural characterisation of candidates 541 and 557 by LC–MS/MS. Abundances were set in relation to the highest peak or signal in the respective extracted ion chromatogram (EIC) or mass spectrum (relative abundance). (a) EIC at *m*/*z* 541 from a neutral loss (−132) measurement of an *E. coli* tRNA hydrolysate. (b) Overlay of QQQ mass spectra at 25.7 min corresponding to the late peak of candidate 541, recorded from an NLS of a hydrolysate of unlabelled (grey) or labelled (^13^C = red, ^15^N = blue, ^34^S = yellow) *E. coli* tRNA. (c) QQQ NLS mass spectrum for the peak at 25.7 min, recorded from hydrolysed *E. coli* tRNA isolated from a ^15^N-labelled culture that was supplemented with ^14^N-uridine and displayed for candidate 541. (d) EIC at *m*/*z* 557 from a neutral loss (−132) measurement of an *E. coli* tRNA hydrolysate. (e) Overlay of QQQ mass spectra at 25.7 min corresponding to the late peak of candidate 557, recorded and displayed as described in (b). (f) QQQ NLS mass spectrum for the peak at 25.7 min for the sample described in c, displayed for candidate 557. (g) QQQ fragmentation mass spectrum for the precursor *m*/*z* 409 at 25.7 min, resulting from the compound with *m*/*z* 541 after in-source fragmentation (pseudo-MS^3^ scan) and illustrating the potential loss of another ribosyl moiety (h) Scheme to illustrate the different product ions occurring in QQQ fragmentation spectra of candidates 541 and 557. (i) QQQ fragmentation mass spectrum for the precursor *m*/*z* 425 at 25.7 min, resulting from the compound with *m*/*z* 557 after in-source fragmentation, similarly to (g), illustrating the potential loss of another ribosyl moiety.

**Fig. 2:**
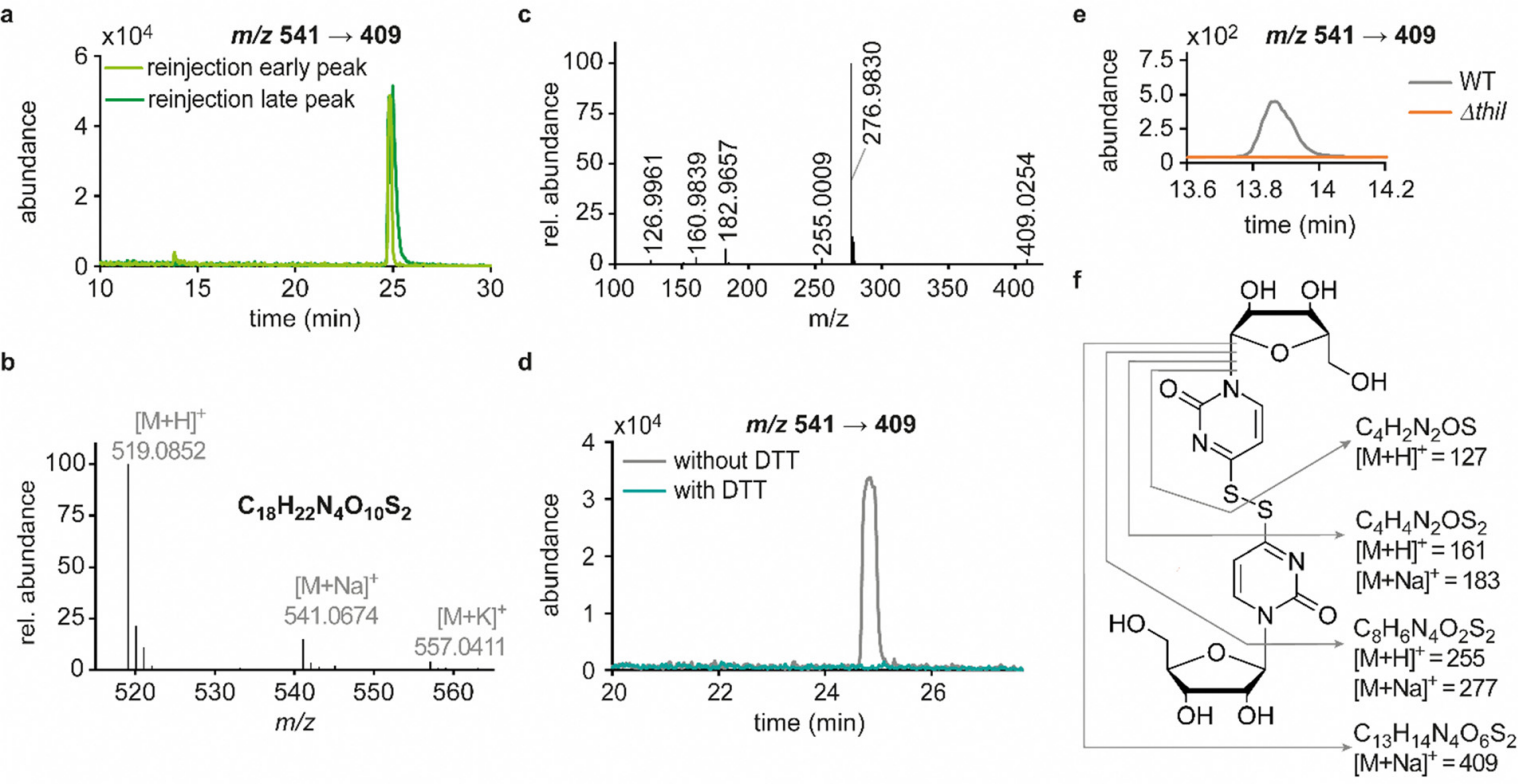
Elucidation of structural details of candidate 541. Abundances were set in relation to the highest peak or signal in the respective extracted ion chromatogram (EIC) or mass spectrum (relative abundance). (a) Merged extracted ion chromatograms (*m*/*z* 541 → 409) for reinjection of the separately collected early (light green) and the late (dark green) peak. (b) Mass spectrum and sum formula calculated for the species of interest from the exact mass detected by HRMS analysis of digested tRNA enriched for candidates 541 and 557. (c) HRMS fragmentation pattern of candidate 541. Sum formulas calculated from exact masses are displayed in panel f of this figure. (d) Merged EICs for the mass transition *m*/*z* 541 → 409 in a QQQ measurement of hydrolysed *E. coli* tRNA as an untreated reference sample (grey) and a sample of collected candidate 541 after dithiothreitol (DTT) treatment (teal). (e) Occurrence of candidate 541 (early RT) in a QQQ measurement of tRNA isolated from the Keio wild type (WT) strain and the thiouridine-related knockout strain *ΔthiI*. (f) Structure proposal for candidate 541 with product ions and their sum formulas being assigned within this structure.

In previous studies we established an NLS method for the highly sensitive identification of potentially new ribonucleoside structures, using a triple quadrupole (QQQ) mass spectrometer coupled to an LC system. Accordingly, we exploited the predominant fragmentation of the vast majority of nucleoside compounds at the *N*-glycosidic bond which leads to the (neutral) loss of the ribose moiety and is accompanied by a mass shift of −132 Da.^11,34^ In order to account for bulky hypermodifications, we set the upper mass limit to an *m*/*z* value of 620 Da. After several filtering steps, the most promising candidates, named according to their mass-to-charge ratios, were subjected to further characterisation, which included several types of MS experiments. While the QQQ-NLS method was used to screen for nucleoside species (*vide supra*) and to determine mass shifts in labelling experiments, we also used the QQQ in a multistage fragmentation approach, a so-called pseudo–MS^3^ scan, by combining a product ion scan with increased fragmentor voltages, in order to induce in-source fragmentation at the most labile bond. The fragmentation patterns obtained from such experiments can provide important information on certain functional groups within an unknown structure. Another powerful tool in the course of the characterisation of candidate structures was high-resolution mass spectrometry (HRMS), which provided exact masses and therefore allowed the calculation of potential ion formulas which can be transferred into potential structure proposals. Of note, due to sensitivity issues, both the QQQ-pseudo MS^3^ scan and the HRMS experiments require higher input of starting material than the initial NLS. A practical solution consisted in collecting fractions of candidates and pooling them after evaporation of the eluate to obtain enriched samples.^11^ At the onset of the present study, within the data set of potentially new ribonucleoside structures, we noticed an early eluting peak at 14.6 min and a later eluting peak at 25.7 min in our reversed-phase chromatography which both were characterised by the simultaneous detection of the same two ions with a mass-to-charge ratio of 541 and 557, respectively (Fig. 1a and d). The exact retention times varied according to the type of experiment, given the use of differentially configured instrumentation (*e.g.* QQQ and HRMS), reversed-phase columns and associated gradients. Hence, for the sake of simplicity, these are referred to as the early and the late peak in the following; methodological details on each experiment are provided in the experimental section. Interestingly, further characterisation of the candidates 541 and 557 by stable isotope labelling revealed identical composition, *i.e.* 18 carbon atoms, four nitrogen atoms and two sulphur atoms, not only for the late and the early peak of each candidate individually but also for both candidates (Fig. 1b, e and Fig. S1a, d, ESI†). On one hand this indicated that the different elution times might be related to different conformations of the same structure, but on the other hand this also drew our attention to a possible relation between the candidates 541 and 557. Typically, the use of NH_4_OAc for precipitation of nucleic acids during sample preparation efficiently minimises adduct formation of nucleoside species and alkali ions during the MS analysis. However, in this case the mass difference of 16 between candidates 541 and 557 demanded to consider the possibility of an adduct formation with the alkaline metal ions Na^+^ and K^+^. Going back to the NLS dataset, a closer inspection did indeed reveal a minute peak of *m*/*z* 519, corresponding to the related H^+^ species at both retention times (Fig. S2, ESI†). Further characteristics shared among the four species were found in stable isotope labelling, specifically during an inverse feeding approach. Here, supplementation of a ^15^N-labelled *E. coli* culture with ^14^N-uridine, identified a pyrimidine species as the underlying nucleoside for all candidates (Fig. 1c, f and Fig. S1b, e, ESI†). Of note, due to the intertwined metabolic pathways yielding cytidine and uridine, it is not possible to distinguish between the two pyrimidine species within the used approach. In a next step, we collected fractions of the eluate around the early retention time of the candidates for enrichment and reinjected this sample for a pseudo–MS^3^ scan. For both candidates, the fragmentation spectra pointed to the loss of a second ribose moiety after the initial loss of the ribose during in-source fragmentation (Fig. 1g–i), further substantiating the assumed relation between candidates 541 and 557. In combination with their comparatively high number of carbon atoms, these findings indicated a potentially dimeric character (Fig. 1h) for both candidates which, due to the limited number of nitrogen atoms, increased the plausibility of a uridine-based dimer. Another intriguing observation during the pseudo-MS^3^ experiment was the fact that reinjection of material collected from either early or late peak resulted in renewed occurrence of two peaks, between which the later was predominant (Fig. S1c and f, ESI†). Given the previously described evidence for a relation of both species with the same *m*/*z*, this finding strongly implied that these presumably different isomers of the same structure might be interconvertible conformers. These findings were recapitulated in different LC–MS settings and corresponding experiments (Fig. 2a (541) and Fig. S3, ESI† (557)), and thus demonstrated that the early and the late eluting compounds are indeed related interconvertible species, presumably conformers. Given that the late peak was consistently predominant in all reinjection experiments, it represents the more stable form under the pertinent experimental conditions. Collected early fractions were also submitted to analysis by high-resolution mass spectrometry (HRMS), and the ensuing calculation of complete sum formulas and thereby unravelled candidates 541 and 557 to be sodium and potassium adducts of a compound with *m*/*z* 519 (Fig. 2b). Interestingly, there was a very pronounced difference in the distribution of protonated *versus* adduct species when comparing analyses from the HRMS instrument (Fig. 2b) and the QQQ instrument (Fig. S2, ESI†) which is most likely a system specific phenomenon but might also be traced to smallest changes in different batches of the mobile phases. Since QQQ measurements were used for further MS analyses and both candidates were shown to be adducts of the same compound with the sodium adduct being the most abundant species during QQQ analysis, further characterisation experiments are representatively shown for this candidate.

**Fig. 3:**
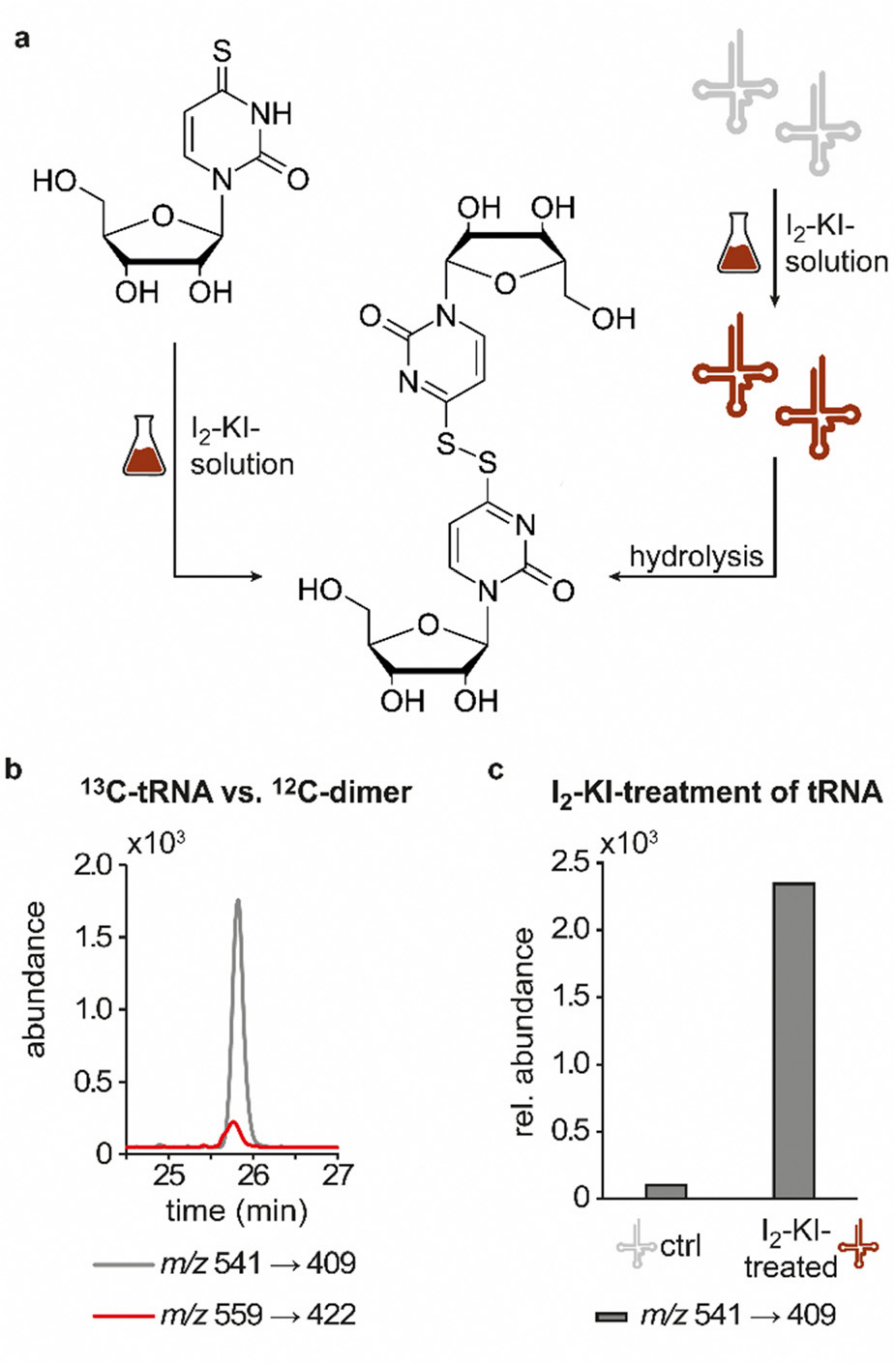
Structural confirmation of the disulfide dimer. (a) Scheme displaying the synthesis of the disulfide dimer *via* treatment of 4-thiouridine with I_2_-KI-solution (left) and induced formation of the disulfide dimer in tRNA *via* its treatment with I_2_-KI-solution with release of the dinucleoside after hydrolysis (right). (b) QQQ LC–MS/MS analysis of a digested ^13^C-labelled tRNA sample (red) spiked with the synthetic ^12^C-dimer (grey). An overlay of the extracted ion chromatograms at a mass transition of *m*/*z* 541 → 409 and *m*/*z* 559 → 422 is shown. (c) Occurrence of candidate 541 in a QQQ measurement of commercial tRNA as an untreated control (ctrl) sample and commercial tRNA treated with I_2_-KI-solution. The abundance of candidate 541 was normalised to the UV-signal of adenosine in the respective sample and the control sample set to 100%.

In line with the earlier observation of two consecutive ribose losses in the QQQ fragmentation spectrum for candidate 541, its behaviour during HRMS fragmentation (Fig. 2c) further substantiated the presumed dimeric character since the sum formula calculated for the main fragment (*m*/*z* = 276.9830, C_13_H_7_N_4_O_2_S_2_^+^) corresponded to the loss of a second ribosyl moiety. Moreover, the calculated sum formulas of other fragments, which mostly occurred as both the sodium adduct and the protonated species, indicated that even with progressing fragmentation the two sulphur atoms remained within the structure. Consequently, we considered a uridine-derived dimer, linked *via* a disulfide, a plausible structure proposal. In keeping with this presumed structure, collected fractions, upon treatment with dithiothreitol (DTT) did not show any more signals for either of the four initial candidates (Fig. 2d). Thus, in the course of further structural elucidation, the two sulphur atoms came to the fore and their position within the molecule had to be determined. In combination with the elemental composition of the potentially new structure, the modifications 2-thiouridine (s^2^U) and s^4^U were of particular interest and theoretically plausible structures included their homodimers as well as a heterodimer, both linked *via* a disulfide bond. Since s^2^U very rarely occurs as such in physiological conditions but rather appears in the context of complex hypermodifications,^41^ we initially focused on the involvement of s^4^U and investigated the effect of a single gene knockout (KO) of its modifying enzyme ThiI on the levels of candidate 541 in tRNA. Importantly, in tRNA isolated from an *E. coli* ThiI knockout the candidate was completely absent (Fig. 2e) which suggested that the potentially dimeric structure arises from s^4^U as a homodimer consisting of two s^4^U monomers whose respective 4-positions are linked to each other *via* a disulfide. The exact masses of product ions obtained during HRMS fragmentation (Fig. 2c) together with the ion formulas calculated from them were consistent with this structure proposal (Fig. 2f). For validation, we synthesized the s^4^U-dimer by treatment of s^4^U with an I_2_-KI-solution (Fig. 3a), following a protocol by Fox *et al.*,^42^ and spiked a hydrolysate of ^13^C-labelled *E. coli* tRNA with this synthetic (^12^C) dinucleoside, to collate their chromatographic behaviour. Here, we observed perfectly matching retention times of the synthetic dimer and the compound occurring in the *E. coli* tRNA at 25.8 min (Fig. 3b), providing ultimate proof that the structure of candidate 541 is a s^4^U-homodimer linked *via* a disulfide bridge. While in earlier experiments we usually observed the joint occurrence of the early and the late eluting substance with *m*/*z* 541 (Fig. 1a), interestingly, the extracted ion chromatogram for the synthetic compound exclusively showed the late peak. Of note, the absence of the early peak suggests that the molecular mechanism of the I_2_-KI oxidation specifically yields a single conformer, which did not equilibrate before analysis. In general, the appearance of two different conformations of the supposed structure was plausible with regard to an earlier study by Irie *et al.* who reported an asymmetric nature of this disulfide and thus two different conformers.^43^ With regard to the biological importance of these findings it is important to mention that already in the 1960s and 1970s the formation of such a structure in tRNA as a result of its exposure to an I_2_-KI-solution was reported.^44^ In order to test if the disulfide dimers detected back then coincided with the detection of candidate 541, we treated *E. coli* tRNA analogously with an I_2_-KI-solution and analysed a hydrolysate of accordingly treated tRNA by LC–MS. This analysis showed significantly increased levels of candidate 541 (Fig. 3c), again with an exclusive occurrence in the late eluting form. Importantly, although the incubation with I_2_-KI-solution stimulated an increased formation of disulfide dimers, in contrast to the above mentioned earlier studies, we also observed the occurrence of this dimeric nucleoside without further treatment of the tRNA in the present case.

**Fig. 4:**
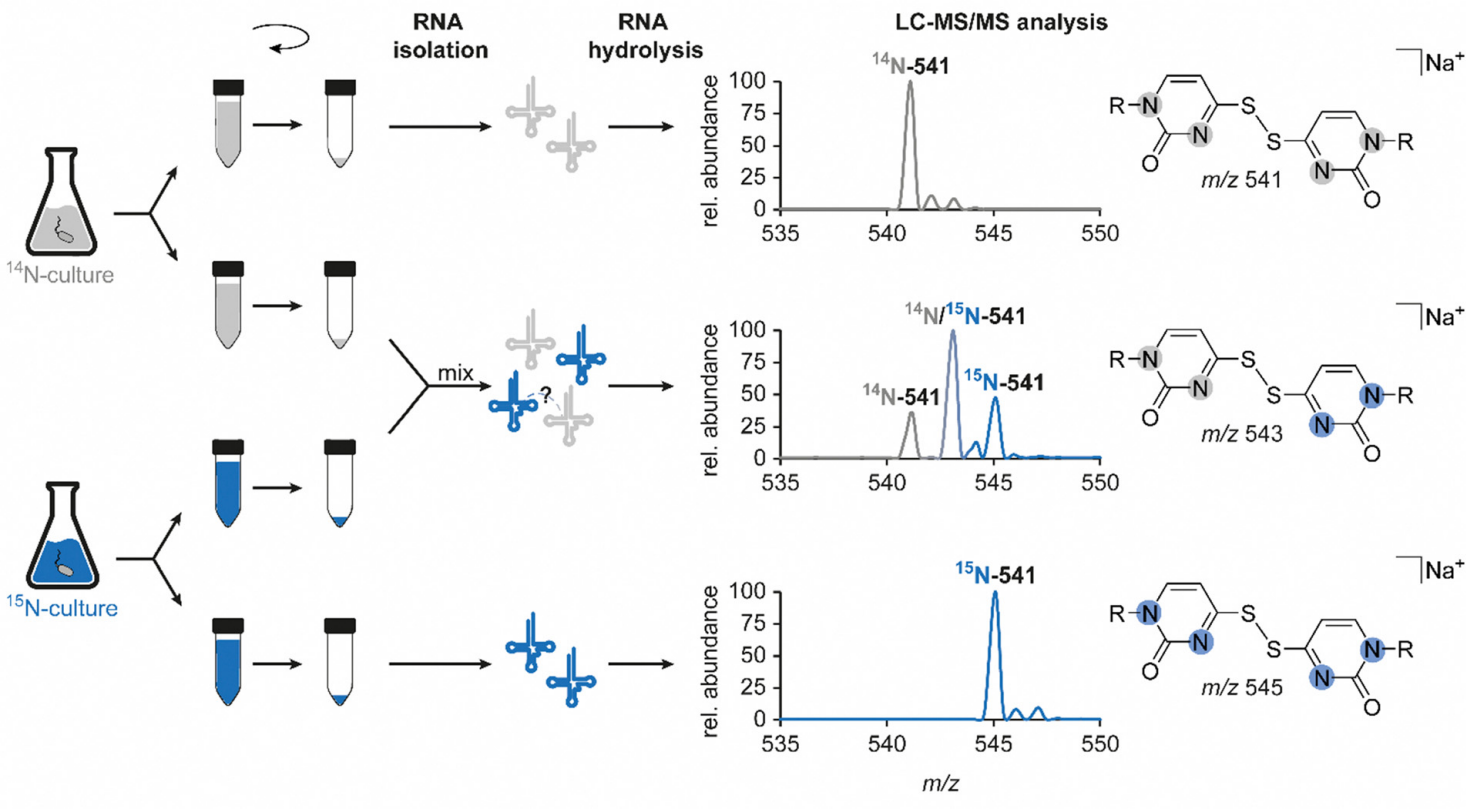
Examination of the authenticity of candidate 541 as a genuine RNA modification. Left: Schematic workflow to examine a potential artificial formation of the dimer during RNA isolation or sample preparation. Right: Results of the LC–MS/MS analysis for respective samples, illustrated by the mass spectrum at the corresponding retention time which was extracted from the neutral loss scan data (−132 Da). ^14^N-541 describes the mass transition of unlabelled candidate 541 (*m*/*z* 541 → 409), ^15^N-541 corresponds to the mass transition of the uniformly ^15^N-labelled compound (*m*/*z* 545 → 413) and the mixed dimer, half unlabelled and half labelled, is illustrated as ^14^N/^15^N-541 (*m*/*z* 543 → 411). Abundances were set in relation to the highest signal in the respective mass spectrum (relative abundance).

In light of previous reports on diverse circumstances affecting the integrity of RNA modifications,^15,16^ we saw a need to examine whether the disulfide dimer was generated *in vivo* or *in vitro*. Therefore, we separately grew an unlabelled and a ^15^N-labelled *E. coli* culture and mixed these samples for the isolation and hydrolysis procedure (Fig. 4). A dimer formed *in vivo* in a biological context, would yield signals corresponding only to compounds containing only a single of the two nitrogen isotopes, *i.e. m*/*z* 541 (unlabelled) and *m*/*z* 545 (^15^N-labelled). In contrast, artificial formation *in vitro* would be expected to yield, in addition to these pure dimers, a signal of an *m*/*z* of 543 mixed dimer, containing equal amounts of ^14^N and ^15^N. Indeed, the occurrence of such a mixed dimer was observed within the described experiment, and at a ratio of 1 : 2 : 1 (Fig. 4) that is compatible with what would be expected for exclusive formation of dimers *in vitro*. This confers the character of an artifact rather than a genuine RNA modification to this non-canonical nucleoside species, described and structurally elucidated within this study. Consequently, the absence of the candidate in the ThiI KO is merely due to the fact that the artificial formation of the dimer requires the presence of s^4^U. Importantly, when we probed for dimer formation after incubation of s^4^U nucleoside under the conditions of hydrolysis, we did not observe any formation of the disulfide (data not shown), indicating that the oxidation occurs on the intact tRNA.

## Conclusions

In search of new RNA nucleoside structures, we detected and structurally elucidated what turned out to be a disulfide-linked dimer of 4-thiouridine in bacterial tRNA. While this structure was not covered in the portfolio of (native) RNA modifications known to date,^3^ early experiments on the oxidation of tRNA concluded the formation of a thiouridine-dimer upon oxidation of tRNA with I_2_-KI-solution. Reported biological consequences of such oxidative tRNA dimerization included loss of acceptor activity.^44–47^ While those early studies attached a biological significance to the dimeric structure, hypothesizing that it might constitute a reversibly deactivated tRNA with regulatory function,^46^ our data (Fig. 4) is fully consistent with oxidative dimerization occurring after RNA isolation. Specifically, any dimerization having occurred *in vivo* would have been expected to shift the ratio of the observed dimers away from the ^14^N–^15^N hybrid species to the homogeneously labelled ^14^N–^14^N and ^15^N–^15^N isotopologues. Given that *in vivo* conditions are typically reductive, and that we have recapitulated reductive cleavage of the dimer by thiols,^44,47^ we conclude that the dimerization took place *in vitro*, presumably during work-up and storage, with the oxidative dimerization likely caused by ambient oxygen. Our data suggests that we are looking at two conformers of the same compound, whose interconversion barrier is high enough that they elute in separate peaks, *i.e.* they do not interconvert during chromatography. Interestingly, oxidation by I_2_-KI exclusively yields the late peak, while air oxidation also produces some of the early peak, presumably because mechanistic details of both oxidation reactions differ. Oxidation in air has been considered a potential problem in protocols exploiting the chemical biology of thiouridines, but no experimental data nor structures were put forward.^36,38,40^ Our present work fills this gap and provides a characterisation of this process that is relevant for, among others, medicinal applications of thio-containing RNAs. Indeed, s^2^U containing mRNA was found to suppress TLR-mediated cytokine emission in dendritic cells.^48^ Given that, in the case at hand, formation of the disulfide linkage occurs *ex vivo*, storage time and conditions of RNA must be considered in general. Even though the consequences in the present case may be limited to a minor falsification of s^4^U levels, oxidative damage to thio-containing RNA during its transport or storage may have dramatic effects on its functionality and will thus have to be subject of thorough studies in the future.

## Experimental section

### RNA preparation

In order to prepare RNA samples, *E. coli* cells (strain MC4100) were cultured in standard M9-medium which contained 6.8 g L^−1^ Na_2_HPO_4_ (Carl Roth, Germany), 3 g L^−1^ KH_2_PO_4_ (Carl Roth, Germany), 0.5 g L^−1^ NaCl (Carl Roth, Germany), 1 g L^−1^ NH_4_Cl (Merck-Millipore, Germany), 2 mM MgSO_4_ (Carl Roth, Germany), 0.1 mM CaCl_2_ (Grüssing GmbH, Germany) and 0.4% glucose (Carl Roth, Germany). For unlabelled RNA, the standard M9-medium was used, whereas single ingredients, namely glucose, NH_4_Cl or MgSO_4_ were replaced with their ^13^C-, ^15^N- or ^34^S-labelled counterparts (all Sigma-Aldrich, Germany), to obtain ^13^C-, ^15^N- or ^34^S-labelled RNA. For differential nucleobase labelling, ^15^N-M9 medium was prepared and the *E. coli* culture was supplemented with either uracil (Carl Roth, Germany), guanine (Carl Roth, Germany) or adenine (Sigma-Aldrich, Germany) in a final concentration of 300 μM.

Respective *E. coli* cultures were grown to an OD_600_ of 1.8, the cells harvested (10 min, 8000 g, 4 °C) and subjected to RNA extraction using TRI reagent® (Sigma-Aldrich, Germany) according to the manufacturer's protocol.

For *E. coli* single gene knockout experiments, we made use of the *E. coli* Keio knockout collection (GE Healthcare (Dharmacon™), United Kingdom) and used the parent strain (BW25113) as well as the *ΔthiI* strain (b0423). The *E. coli* cells were cultured in standard M9-medium and RNA was isolated.

### Oxidation of tRNA *in vitro*

Following the protocol published by Carbon *et al.*, commercial tRNA (Sigma Aldrich, Germany) was dissolved to a concentration of 5 mg mL^−1^ in 0.01 M Tris–HCl (pH 7).^46^ After addition of an equal volume of 1 mM iodine in 0.5% KI-0.01 M Tris–HCl (pH 7), the mixture was incubated on ice for 20 min. Subsequently, the tRNA was precipitated twice by addition of 1/10 volume of 5 mM NH_4_OAc and 2.5 volumes of ice-cold ethanol, respectively.

### Synthesis of the s4U-dimer

The reaction previously described by Fox *et al.* was carried out in a sub-milligram scale using 102.5 μL of an aqueous solution of s^4^U (10 mM), 25 μL Sörensen phosphate buffer (pH 6.8) and 0.5 μL of 1 N iodine solution.^42^ A colourless solid precipitated, the supernatant was discarded after centrifugation and the product was lyophilized. In order to obtain an amount sufficient for NMR analysis, the synthesis and purification procedure were repeated several times and pooled products were dissolved in DMSO-d_6_.

NMR spectra were recorded on a Bruker Avance III spectrometer (Bruker Corporation, USA). Chemical shifts were reported in parts per million (ppm) relative to tetramethylsilane and internally referenced to DMSO-d_6_ (2.50 ppm for ^1^H-NMR and 39.5 ppm for ^13^C-NMR). ^1^H–^1^H COSY, ^1^H–^13^C HSQC and ^1^H–^13^C HMBC spectra provided the basis for signal assignment. Coupling constants *J* are given in Hz and multiplicities were abbreviated as follows: d = doublet, dd = doublet of doublets, dt = doublet of triplets.

^1^H-NMR (600 MHz, DMSO-d_6_): *δ* = 8.53 (d, *J* = 7.1 Hz, 2H, H-6,6′), 6.79 (d, *J* = 7.1 Hz, 2H, H-5,5′), 5.70 (d, *J* = 2.6 Hz, 2H, H-7,7′), 3.98 (dd, *J* = 4.5, 2.6 Hz, 2H, H-8,8′), 3.94 (dd, *J* = 6.9, 4.5 Hz, 2H, H-9,9′), 3.91 (dt, *J* = 6.9, 2.6 Hz, 2H, H-10,10′), 3.74 (dd, *J* = 12.4, 2.6 Hz, 2H, H-11a,11′a), 3.59 (dd, *J* = 12.4, 2.7 Hz, 2H, H-11b,11′b) ppm.

^13^C-NMR (151 MHz, DMSO-d_6_): *δ* = 174.0 (2C, C-4,4′), 152.4 (2C, C-2,2′), 144.3 (2C, C-6,6′), 99.8 (2C, C-5,5′), 90.7 (2C, C-7,7′), 84.1 (2C, C-10,10′), 74.4 (2C, C-8,8′), 68.1 (2C, C-9,9′), 59.4 (2C, C-11,11′) ppm.

### RNA hydrolysis to nucleoside level

Prior to its LC–MS/MS analysis, total tRNA (up to 10 μg) was digested to nucleoside level by incubation with the following enzymes and deaminase inhibitors in 25 mM ammonium acetate (pH 7.5; Sigma-Aldrich, Germany) overnight at 37 °C: 0.6 U nuclease P1 from *P. citrinum* (Sigma-Aldrich, Germany), 0.2 U snake venom phosphodiesterase from *C. adamanteus* (Worthington, USA), 2 U FastAP (Thermo Fisher Scientific, Germany), 10 U benzonase (Sigma-Aldrich, Germany), 200 ng Pentostatin (Sigma-Aldrich, Germany) and 500 ng Tetrahydrouridine (Merck-Millipore, Germany).

### LC–MS experiments

For all LC–MS/MS measurements an Agilent 1260 Infinity (II) series HPLC in combination with either an Agilent 6460A or 6470B triple quadrupole (QQQ) mass spectrometer or a high-resolution Agilent 6545 quadrupole time-of-flight (Q-ToF) mass spectrometer, each of them equipped with an Agilent Jet Stream electrospray ionisation source (ESI), was used (all devices: Agilent Technologies, Germany). Initially and mostly, a YMC-Triart C8 column (150 × 3 mm, 3 μm, 120 Å; YMC Europe GmbH, Germany) at a flow of 0.35 mL min^−1^ and a temperature of 35 °C was used for separation, which was later on replaced by a Synergi Fusion RP-C18 column (250 × 2.0 mm, 4 μM, 80 Å; Phenomenex, Germany) that was employed under the same conditions and whose use is stated accordingly. The mobile phase consisted of 5 mM ammonium acetate buffer (pH 5.3; solvent A) and LC–MS grade acetonitrile (solvent B; Honeywell). Starting at 100% solvent A, in a first step of the gradient, solvent B was linearly increased to 10% at 20 min. After another linear increase of solvent B to 100% at 50 min, which was held for 3 min, the initial conditions were restored within 7 min and the column re-equilibrated at 100% solvent A for 5 min. With regard to the use of different instrumentation or columns, it is important to mention, that the identity of the candidates was ensured by comparing the results of stable isotope labelled samples and the retention time relative to known modifications. The LC systems contained a diode array detector (DAD) which allowed to monitor the main nucleosides at a wavelength of 254 nm (UV trace). All mass spectrometers were operated in the positive ion mode and details on respective source parameters are provided in Table 1.

**Table 1:** ESI settings of the different mass spectrometers

Parameters	6460A (QQQ)	6470B (QQQ)	6545 (Q-ToF)
Gas temperature	350	300	325
Gas flow (L min^−1^)	5	7	10
Nebulizer pressure (psi)	35	60	33
Sheath gas temperature (°C)	350	400	325
Sheath gas flow (L min^−1^)	12	12	10
Capillary voltage (V)	3500	3000	3500

#### Qualitative analysis

Unless otherwise stated, qualitative LC–MS/MS analysis was performed with the 6460A QQQ and included identification of candidate 541 and 557 as potentially new ribonucleosides as previously described and the examination of their mass shifts in stable isotope labelled samples.^11,34^ Therefore, 10 μg of hydrolysed RNA sample were separated using the C8 column and the Agilent MassHunter software (version B.05.00) was employed in the NLS mode. In the present case mostly a neutral loss of 132 Da, corresponding to the loss of the ribose moiety, was programmed into the method. Importantly, when the sample of interest was ^13^C-labelled, the neutral loss had to be adjusted to 137 Da, corresponding to a ^13^C-labelled ribose moiety. The variable parameters of the NLS settings of the 6460A QQQ were set to the following values: fragmentor voltage 80 V, collision energy 15 eV, cell accelerator voltage 2 V, scan time 555 ms and mass range *m*/*z* 460–620.

In order to compare the chromatographic behaviour of the candidate occurring in RNA and the synthetic s^4^U-dimer, 5 μg of hydrolysed ^13^C-labelled RNA and 1 pmol of synthetic s^4^U-dimer were co-injected and separated on the C18 column. During this analysis the 6460A QQQ was used with the Agilent MassHunter software (version B.05.00) being employed in the dynamic multiple reaction monitoring (dMRM) mode. The mass transitions for the predefined nucleosides were as follows: 541-^12^C (*m*/*z* 541 → 409) and 541-^13^C (*m*/*z* 559 → 422). Further dMRM settings were a fragmentor voltage of 80 V, a collision energy of 15 eV and a cell accelerator voltage of 2 V.

For analysis of the oxidized RNA samples obtained after I_2_-KI-treatment of tRNA, 5 μg of hydrolysed RNA sample were injected into the LC–MS system. Here, the C18 column was used for separation and the 6470B QQQ (Agilent MassHunter software, version 10.0) was used in the dMRM mode, detecting the mass transition of candidate 541 (*m*/*z* 541 → 409). Using the Agilent MassHunter Qualitative Analysis software (version 10.0), the peaks of interest were integrated and the total area under the curve (abundance) resulting from addition of the respective area under the curve of the early and the late peak was normalised to the peak area of adenosine in this sample, to account for different amounts of RNA.

Fragmentation patterns of candidates 541 and 557 were recorded in so-called pseudo-MS^3^ scans by using the 6460A QQQ and the Agilent MassHunter software (version B.05.00) in the product ion scan mode with an increased fragmentor voltage of 200 V. The latter leads to an in-source fragmentation of the nucleosides yielding nucleobases in the MS^1^ detector which filters for the programmed precursor ions *m*/*z* 409 and *m*/*z* 425. In the collision cell the nucleobase was subjected to further fragmentation by application of a collision energy of 35 eV (candidate 541) and a collision energy of 20 eV (candidate 557), resulting in a product ion spectrum. The QQQ settings were similar to the NLS except for the scan time which was changed to 500 ms and the MS^2^ scan range which was changed to *m*/*z* 15-220. Due to sensitivity issues, the candidates were enriched by collecting the fraction of 50 μg hydrolysed and chromatographically separated *E. coli* RNA around the elution time of the early peak five times (*vide infra*). After evaporation of the solvent, the dry residues of the fractions were pooled in MilliQ water.

#### Interconversion: reinjection of collected samples

The two peaks of candidate 541 were collected separately during separation of 50 μg hydrolysed *E. coli* tRNA on the C8 column by starting the collection 0.2 min before the exact retention time and continuing the collection until 1 min after the exact retention time. Subsequently, the solvent was evaporated and the dry residues of these fractions were resolved in MilliQ water. These samples were then reinjected into the LC–MS system and analysed by MRM for the mass transition of the candidates 541 (*m*/*z* 541 → 409) and 557 (*m*/*z* 557 → 425). The data was analysed using the Agilent MassHunter Qualitative Analysis software (version B.05.00).

#### DTT treatment of candidate 541

A sample of five times enriched candidate 541 was treated with dithiothreitol (DTT) (Thermo Fisher Scientific, Germany) at a final concentration of 100 mM for 2 h at 25 °C. Subsequently, the treated sample and an untreated reference sample of hydrolysed commercial *E. coli* tRNA were analysed in an MRM measurement as described above.

#### High-resolution mass spectrometry analysis

HRMS was performed on an Agilent 6545 Q-ToF mass spectrometer and the C8 column was used for separation. Due to sensitivity issues, the candidates were enriched by collecting the fraction of 50 μg hydrolysed and chromatographically separated *E. coli* tRNA around the elution time of the early peak ten times. After evaporation of the solvent, the dry residues of the fractions were pooled in MilliQ water.

In order to identify the exact masses of candidates 541 and 557, the Agilent MassHunter software (version 10.0) was employed in the MS mode from *m*/*z* 100 to *m*/*z* 1000 at a scan rate of 1 spectrum per s. Based on the experimentally detected mass, the sum formula calculator within the software allowed calculation of the exact mass and the corresponding sum formula.

HRMS (ESI): *m*/*z* calcd for C_18_H_22_N_4_O_10_S_2_: 519.0850 [M + H]^+^, found: 519.0852; *m*/*z* calcd for C_18_H_22_N_4_O_10_S_2_: 541.0670 [M + Na]^+^, found: 541.0674; *m*/*z* calcd for C_18_H_22_N_4_O_10_S_2_: 557.0409 [M + K]^+^, found: 557.0411.

The exact masses of the fragments (identified by pseudo-MS^3^ analysis) were determined by use of the Agilent MassHunter software (version 10.0) in the targeted MS^2^ mode. The precursor ion used in this analysis was *m*/*z* 541.0700 and the centroid mode was used for data acquisition with the following method settings: MS *m*/*z* 100–1000, MS scan rate 1 spectrum per s, MS/MS *m*/*z* 30–500, MS/MS scan rate 1 spectrum per s, max time between MS 5 s and use of fixed collision energies (35 eV).

HRMS (ESI): *m*/*z* calcd for C_4_H_2_N_2_OS: 126.9961 [M + H]^+^, found: 126.9964; *m*/*z* calcd for C_4_H_4_N_2_OS_2_: 160.9838 [M + H]^+^, found: 160.9839; *m*/*z* calcd for C_4_H_4_N_2_OS_2_: 182.9657 [M + Na]^+^, found: 182.9657; *m*/*z* calcd for C_8_H_6_N_4_O_2_S_2_: 255.0005 [M + H]^+^, found: 255.0009; *m*/*z* calcd for C_8_H_6_N_4_O_2_S_2_: 276.9824 [M + Na]^+^, found: 276.9830; *m*/*z* calcd for C_13_H_14_N_4_O_6_S_2_: 409.0247 [M + Na]^+^, found: 409.0254.

## Author contributions

L. Bessler performed the majority of the experimental work except for NMR analysis which was performed by J. Groß and T. Opatz as well as HRMS measurements which were performed by C. J. Kampf. All authors discussed the results. L. Bessler and M. Helm wrote the manuscript which was reviewed and edited by all authors. M. Helm designed and supervised the work.

## Conflicts of interest

Mark Helm is a consultant for Moderna Inc.

## Supplementary Material

CB-005-D3CB00221G-s001
