# Supplementary material for: Reversible oxidative dimerization of 4-thiouridines in tRNA isolates
Source: RSC Chem Biol. 2024 Feb 1;5(3):216–24. doi: 10.1039/d3cb00221g (PMC10915967; doi:10.1039/d3cb00221g)
Supplement: CB-005-D3CB00221G-s001 [file CB-005-D3CB00221G-s001.pdf]

Electronic Supplementary Material (ESI) for RSC Chemical Biology.
This journal is © The Royal Society of Chemistry 2024

Reversible oxidative dimerization of 4-thiouridines in tRNA isolates

Larissa Bessler, [a] Jonathan Groß [b], Christopher J. Kampf [b], Till Opatz [b] and Mark Helm *[a]

[a.] *Institute of Pharmaceutical and Biomedical Sciences, Johannes Gutenberg University Mainz, Staudingerweg 5, 55128 Mainz, Germany.*
E-mail: mhelm@uni-mainz.de
[b.] *Department of Chemistry, Johannes Gutenberg University Mainz, Duesbergweg 10–14, 55128 Mainz, Germany.*

Supplementary Figures

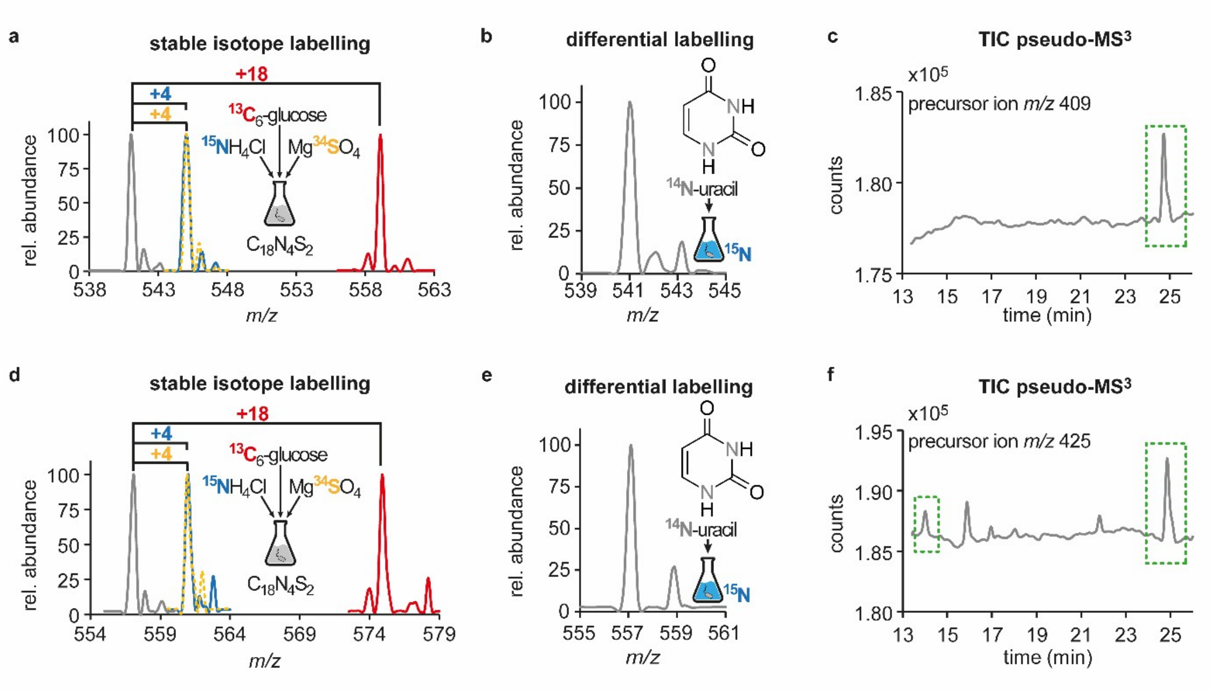

Supplemental Figure S1: Abundances were set in relation to the highest peak in the mass spectrum (relative abundance). **a)** Overlay of QQQ mass spectra at 14.6 min corresponding to the early peak of candidate 541, recorded from an NLS of a hydrolysate of unlabelled (grey) or labelled (^{13}C = red, ^{15}N = blue, ^{34}S = yellow) *E. coli* tRNA. **b)** QQQ NLS mass spectrum for the peak at 14.6 min, recorded from hydrolysed *E. coli* tRNA isolated from a ^{15}N-labelled culture that was supplemented with ^{14}N-uridine and displayed for candidate 541. **c)** Total ion chromatogram (TIC) obtained from the product ion scan for the precursor ion with *m/z* 409, resulting from in-source fragmentation of candidate 541. For this analysis, a sample of hydrolysed *E. coli* tRNA, enriched for candidate 541 (collection of a fraction in the time frame of the early peak) was used. The dotted frame in green highlights the signal corresponding to candidate 541. **d)** Overlay of QQQ mass spectra at 14.6 min corresponding to the early peak of candidate 557, recorded and displayed as described in a). **e)** QQQ NLS mass spectrum for the peak at 14.6 min for the sample described in b), displayed for candidate 557. **f)** TIC obtained from the product ion scan for the precursor ion with *m/z* 425, resulting from in-source fragmentation of candidate 557. A sample of hydrolysed *E. coli* tRNA, enriched for candidate 557 (collection of a fraction in the time frame of the early peak) was used. The dotted frame in green highlights the signals corresponding to candidate 557.

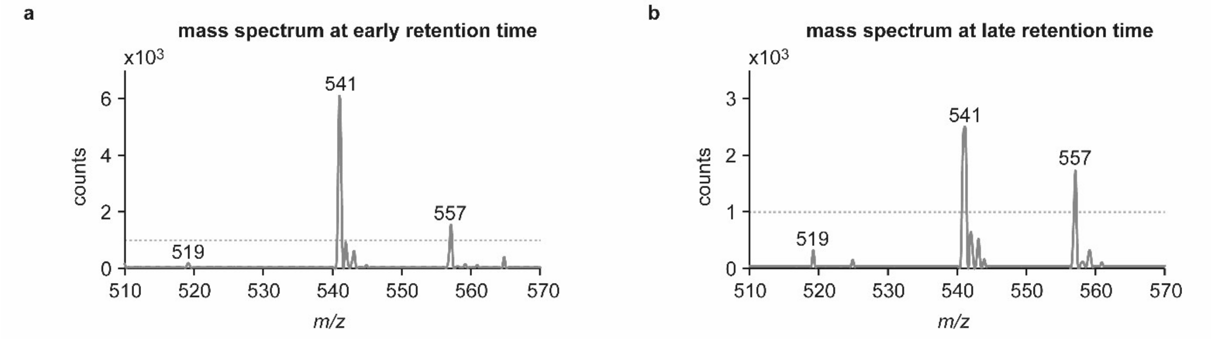

Supplemental Figure S2: QQQ NLS mass spectra of the candidate at the early retention time 14.6 min (**a**) and the late retention time 25.7 min (**b**), recorded from an NLS of unlabelled *E. coli* tRNA hydrolysate. The dashed line marks the threshold of 10^3 which was used as a filter criterion during generation of the dataset of potentially new ribonucleosides.

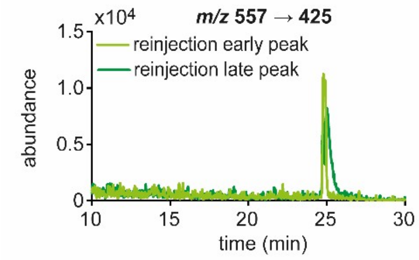

Supplemental Figure S3: Merged extracted ion chromatograms (*m/z* 557 → 425) for reinjection of the separately collected early (light green) and the late (dark green) peak.

NMR Spectra

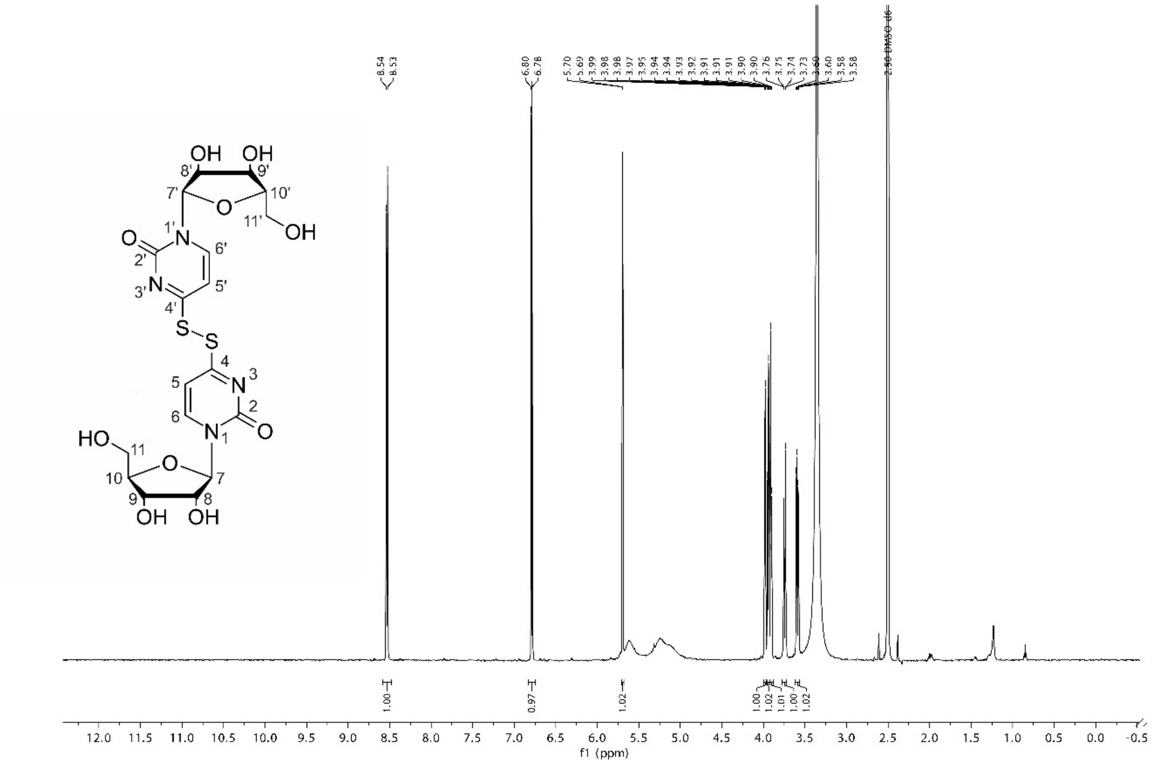

^{1}H-NMR spectrum of the s^4U dimer (600 MHz, DMSO-d_6).

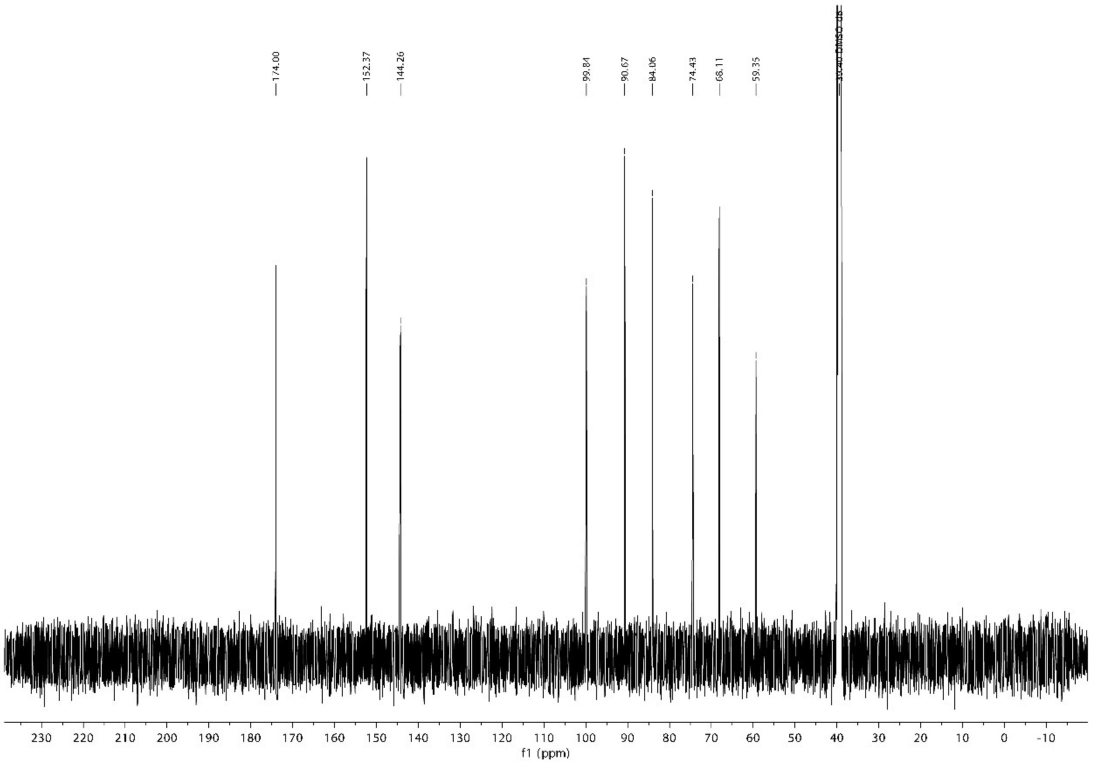

^{13}C-NMR spectrum of the s^4U dimer (151 MHz, DMSO-d_6).
